# Sustained IP3-linked Ca^2+^ signaling promotes progression of triple negative breast cancer cells by regulating fatty acid metabolism

**DOI:** 10.3389/fcell.2023.1071037

**Published:** 2023-03-13

**Authors:** Riccardo Filadi, Agnese De Mario, Matteo Audano, Patrizia Romani, Silvia Pedretti, Cesar Cardenas, Sirio Dupont, Cristina Mammucari, Nico Mitro, Paola Pizzo

**Affiliations:** ^1^ Neuroscience Institute, National Research Council (CNR), Padua, Italy; ^2^ Department of Biomedical Sciences, University of Padova, Padua, Italy; ^3^ Department of Pharmacological and Biomolecular Sciences, University of Milan, Milan, Italy; ^4^ Department of Molecular Medicine (DMM), University of Padova, Padua, Italy; ^5^ Faculty of Sciences, Universidad Mayor, Center for Integrative Biology, Santiago, Chile; ^6^ Geroscience Center for Brain Health and Metabolism, Santiago, Chile; ^7^ Buck Institute for Research on Aging, Novato, CA, United States; ^8^ Department of Chemistry and Biochemistry, University of California, Santa Barbara, CA, United States; ^9^ Myology Center (CIR-Myo), University of Padova, Padua, Italy; ^10^ Department of Experimental Oncology, European Institute of Oncology IRCCS, Milan, Italy

**Keywords:** IP3, Ca^2+^, breast cancer, TNBC, mitochondria, MCU, fatty acids, acylcarnitine

## Abstract

Rewiring of mitochondrial metabolism has been described in different cancers as a key step for their progression. Calcium (Ca^2+^) signaling regulates mitochondrial function and is known to be altered in several malignancies, including triple negative breast cancer (TNBC). However, whether and how the alterations in Ca^2+^ signaling contribute to metabolic changes in TNBC has not been elucidated. Here, we found that TNBC cells display frequent, spontaneous inositol 1,4,5-trisphosphate (IP3)-dependent Ca^2+^ oscillations, which are sensed by mitochondria. By combining genetic, pharmacologic and metabolomics approaches, we associated this pathway with the regulation of fatty acid (FA) metabolism. Moreover, we demonstrated that these signaling routes promote TNBC cell migration *in vitro*, suggesting they might be explored to identify potential therapeutic targets.

## 1 Introduction

Cancer cells require a considerable amount of energy to fulfill processes such as cell proliferation, migration and metastasis. ATP is the major cellular energy currency and, in most cell types, it is predominantly synthesized through oxidative phosphorylation (OXPHOS) by mitochondria. Nevertheless, Otto Warburg first suggested that mitochondrial impairment, compensated by glycolysis upregulation, might be crucial to cancer onset (Warburg, 1956). The reason underlying this apparently inefficient metabolic switch remained elusive until recently (Heiden et al., 2009), when it has emerged that the rewiring of specific mitochondrial functions is essential for proliferation and invasiveness of certain cancer cells (De Berardinis and Chandel, 2016). In particular, different metabolic intermediates from the tricarboxylic acid (TCA) cycle fuel anaplerotic reactions, such as lipid, nucleic acid and protein biosynthesis (Ahn and Metallo, 2015; Boroughs and DeBerardinis, 2015); furthermore, mitochondrial activity is associated with the generation of reactive oxygen species (ROS) (Sullivan and Chandel, 2014). These reactions and signaling molecules are essential for proliferation, as well as migration, of different malignant cells (Sullivan and Chandel, 2014; Ahn and Metallo, 2015; Boroughs and DeBerardinis, 2015).

Remarkably, several mitochondrial activities are regulated by Ca^2+^, both in the matrix and in the intermembrane space (Denton, 2009; Rossi et al., 2019). Different oncogenes limit an excessive mitochondrial Ca^2+^ uptake, thus preventing apoptotic signals and favouring cancer cell survival (Rimessi et al., 2020). However, recent studies reappraised the classical paradigm whereby cancer cells inevitably dampen mitochondrial Ca^2+^ uptake to escape apoptosis, actually suggesting that addiction to sustained mitochondrial Ca^2+^ levels may be a feature of certain cancer cells (Cardenas et al., 2016, 2020; Tosatto et al., 2016; Bittremieux et al., 2018). On this line, pharmacological inhibition of the constitutive Ca^2+^ transfer from endoplasmic reticulum (ER) to mitochondria has been reported to affect the viability of several cancer cell lines (Cardenas et al., 2016, 2020). Moreover, the ablation of the mitochondrial Ca^2+^ uniporter (MCU, *i.e.*, the channel mediating mitochondrial Ca^2+^ uptake) deeply affects the invasiveness of TNBC cells (Tosatto et al., 2016). Importantly, however, though it has been suggested that TCA cycle (Cardenas et al., 2016) and ROS signaling (Tosatto et al., 2016) are key targets, the mechanisms underlying the sustained mitochondrial Ca^2+^ uptake and its metabolic significance have not been fully elucidated.

## 2 Materials and methods

### 2.1 Cell culture, plasmids, and transfection

MDA-MB-231 CTRL and MDA-MB-231 MCU-KO cell clones were previously described (Tosatto et al., 2016) and maintained in DMEM/F12 medium (Gibco 31330038) supplemented with 10% FCS, 100 U/ml penicillin and 0.1 mg/ml streptomycin. MCF10A cells were maintained in DMEM/F12 supplemented with 5% horse serum, Cholera toxin (0.1 nM, Sigma Aldrich C8052), hEGF (20 ng/ml, Peprotech AF-100-15), insulin (1X, Sigma-Aldrich I9278), hydrocortisone (500 ng/ml, Sigma-Aldrich H0396), 100 U/ml penicillin and 0.1 mg/ml streptomycin. MDA-MD-468 and BT-549 cells were maintained in DMEM medium (Sigma-Aldrich D5671) supplemented with 10% FCS, 2 mM glutamine, 100 U/ml penicillin and 0.1 mg/ml streptomycin. Cells were grown in a humidified Heraeus incubator at 37°C, with 5% CO_2_. For a better comparison, all cell types were seeded 24–48 h before the experiments in DMEM/F12 medium supplemented with 10% FCS, 100 U/ml penicillin and 0.1 mg/ml streptomycin. Transfection was performed 24 h before the experiments by TransIT-LT1 (Mirus Bio) following manufacturer instructions. The cDNA for nuclear and mitochondrial ATeam1.03 (Imamura et al., 2009), GFP-PHD (Hirose et al., 1999), and mitochondrial 4 mt-GCamp6f (Patron et al., 2014) were previously described.

### 2.2 Protein extraction and western blot analysis

Cells were washed with PBS and lysed in RIPA buffer (50 mM Tris, 150 mM NaCl, 1% Triton X-100, 0.5% deoxycholic acid, 0.1% SDS, protease and phosphatase inhibitor cocktails (Roche, 11836170001 and 4906845001, respectively, pH 7.5). The lysates were incubated on ice for 30 min. Supernatant was collected after spun down at 13,000 rcf for 15 min at 4°C. 45 μg of protein were separated by SDS-PAGE, blotted and probed with the following antibodies: α-IP3R-1 (ThermoFisher Scientific, PA3-901A), α-actin (A4700, Sigma-Aldrich), α-IP3R-3 (BD Biosciences, 610312), α-CPT1A (Proteintech, 15184-1-AP). ECL (Amersham, GE Healthcare) was used to detect chemiluminescent signal using an Uvitec Mini HD9 apparatus (Eppendorf). The intensity of the bands was calculated by ImageJ (NIH).

### 2.3 Ca^2+^, ATP and GFP-PHD measurements

For Ca^2+^ measurements by Fura-2, 1 × 10^5^ cells were seeded on 18 mm coverslips and grown for 24 h in DMEM/F12 supplemented as described above. On the day of experiments, cells were incubated with 1 µM Fura-2/AM, 0.02% w/V pluronic F-127 and 200 µM sulfinpyrazone in mKRB medium (in mM: 140 NaCI, 2.8 KCI, 2 MgCl_2_, 10 HEPES, 1 CaCl_2_, 10 glucose, pH 7.4 at 37°C) for 30 min at 37°C, washed 3x with mKRB and coverslips were mounted on a chamber with mKRB (either with CaCl_2_ 1 mM or with EGTA 0.5 mM, as indicated). Cells were visualized by a ×40 ultraviolet-permeable objective (Olympus Biosystems) on an inverted microscope (Zeiss Axiovert 100), exciting at 340 and 380 nm through a monochromator (polychrome V, TILL-Photonics, 200 ms exposure time at each wavelength) and collecting emission fluorescence every 2 s (500–530 nm band-pass filter, Chroma) by a PCO SensiCam QE (Kelheim) camera, controlled by a custom-made software (Roboscope, developed by Catalin Dacian Ciubotaru). The ratio between the two fluorescence intensities (340/380 nm, proportional to [Ca^2+^]) was calculated by tracing specific ROIs. To calculate the % of cells displaying Ca^2+^ oscillations, a threshold (Fura-2 340/380 nm R = 0.35) was imposed, corresponding to the basal 340/380 nm R + 1Dv.Std of MDA-MB-231 CTRL cells. The cells displaying R values >0.35 during the experiments (10 min) were considered as positive.

For evaluation of mitochondrial Ca^2+^ dynamics by 4 mt-GCamp6f, 24 h after transfection (see above) cells were washed with mKRB, mounted on a chamber and bathed in mKRB medium, either containing or not 2% FCS. Imaging was performed on the same microscope describe above for Fura-2, by exciting sequentially at 475 nm (180 ms) and at 410 nm (300 ms). Emission fluorescence was collected in the 500–530 nm range by a band-pass filter (Chroma) every second. After background subtraction, the ratio (proportional to [Ca^2+^]) of the emissions upon 475/410 nm excitations was calculated by tracing specific ROIs corresponding to mitochondrial network.

To evaluate ATP dynamics by ATeam1.03 FRET-based probes, 24 h after transfection cells were washed with mKRB, mounted on a chamber and bathed in mKRB medium. Imaging was performed on a DM6000 inverted microscope (Leica) with a 40× oil objective (HCX Plan Apo, NA 1.25). Excitation light (405 nm, Led Engin #LZ1-00UA00) was filtered at the appropriate wavelength (415–435 nm) through a band-pass filter. Exposure was 200 ms and images were acquired every 5 s. The emitted light was collected through a beam splitter (OES s. r.l, Padua, Italy) (emission filters HQ 480/40M (CFP); HQ 535/30M (YFP); dichroic mirror 515 DCXR). All filters and dichroic were from Chroma Technologies. Images were collected by an IM1.4C cool camera (Jenoptik Optical Systems) controlled by the software Roboscope (see above) and the FRET ratio between YFP and CFP emissions calculated by tracing specific ROIs.

PLC activity by evaluating GFP-PHD subcellular distribution upon IP3-generating stimuli was performed 24 h after transfection on a Thunder Imager 3D Cell Culture (Leica), equipped with a LED8 illumination system (the 475 nm line was used to excite GFP-PHD), a 40x/1.30 oil immersion objective (HC PL Fluotar 340) and with a cool camera (Hamamatsu Flash 4.0 V3). Excitation and emission light was filtered by a GFP-dedicated set of filters (Chroma). Upon IP3-generating cell stimulations, GFP-PHD migrates from the sub-plasma membrane region to the cytosol; therefore, PLC activity was evaluated by measuring the intensity of cytosolic GFP-PHD fluorescent signal (F) normalized for that at the beginning of the experiment (F0), as described (Hirose et al., 1999).

### 2.4 OCR measurements

MDA-MB-231 CTRL cells (2.5 × 10^4^/well) were seeded in XF24 cell culture microplates (Seahorse Bioscience). After 24 h, cells were treated over night with either DMSO, BAPTA-AM (10 µM), U-73343 (2.5 µM) or U-73122 (2.5 µM). The day after, the medium was replaced with 670 μL of mKRB supplemented with 2% FCS, glutamine (2 mM), sodium pyruvate (0.5 mM) and with either DMSO, BAPTA-AM (10 µM), U-73343 (2.5 µM) or U-73122 (2.5 µM). Cells were incubated at 37°C for 30 min; OCR was measured with an XF24 Extracellular Flux Analyzer (Seahorse Bioscience). After OCR baseline measurement, specific drugs were added as indicated (oligomycin-A (1 μg/ml); carbonyl cyanide-4-(trifluoromethoxy)phenylhydrazone (FCCP, 1 μM); rotenone (1 μM) and antimycin-A (1 μM). At the end of the experiments, cells were counted for each treatment (DMSO, BAPTA-AM, U-73343, U-73122) and data normalized to the baseline OCR of the DMSO condition. ATP-linked and maximal OCRs were calculated respectively as (*OCR*_*basal*_ *- OCR*_*Oligomycin-A*_) and (*OCR*_*FCCP*_ *- OCR*_*Rot/AA*_).

### 2.5 Targeted metabolomics analysis

Cells (1 × 10^6^) were grown for 24 h in 10 cm Petri dishes, harvested with trypsin, spun down in DMEM/F12 (800 rcf) for 5 min. Cells were washed 3x in ice-cold PBS, immediately frozen in liquid-N_2_ and stored at −80°C. Metabolomics data were obtained by liquid chromatography coupled to tandem mass spectrometry. We used an API-3500 triple quadrupole mass spectrometer (AB Sciex) coupled with an ExionLC™ AC System (AB Sciex, Framingham, MA, United States). Cells were extracted using a tissue lyser for 1 min at maximum speed in 250 µL of ice-cold methanol:water:acetonitrile 55:25:20 containing [U-^13^C_6_]-glucose 1 ng/μL and [U-^13^C_5_]-glutamine 1 ng/μL as internal standards (Merck Life Science). Lysates were spun at 15,000 g for 15 min at 4°C. Samples were then dried under N_2_ flow at 40°C and resuspended in 125 µL of ice-cold methanol:water:acetonitrile 55:25:20 for subsequent analyses.

Amino acids, their derivatives and biogenic amine quantification were performed through previous derivatization. Briefly, 25 µL out of 125 µL of samples were collected and dried separately under N_2_ flow at 40°C. Dried samples were resuspended in 50 µL of phenyl-isothiocyanate (PITC, Merck Life Science), EtOH, pyridine and water 5%:31.5%:31.5%:31.5% and then incubated for 20 min at RT, dried under N_2_ flow at 40°C for 90 min and finally resuspended in 100 µL of 5 mM ammonium acetate in MeOH:H_2_O 50:50. Quantification of different amino acids was performed by using a C18 column (Biocrates) maintained at 50°C. The mobile phases for positive ion mode analysis were phase A: 0.2% formic acid in water and phase B: 0.2% formic acid in acetonitrile. The gradient was T0: 100% A, T5.5: 5% A and T7: 100% A with a flow rate of 500 μL/min. All metabolites analyzed in the described protocols were previously validated by pure standards, and internal standards were used to check instrument sensitivity.

Quantification of energy metabolites and cofactors was performed by using a cyanophase LUNA column (50 mm × 4.6 mm, 5 μm; Phenomenex) by a 5.5 min run in negative ion mode with two separated runs. Protocol A: mobile phase A was water, phase B was 2 mM ammonium acetate in MeOH, and the gradient was 10% A and 90% B for the analysis, with a flow rate of 500 μL/min. Protocol B: mobile phase A was water, phase B was 2 mM ammonium acetate in MeOH, and the gradient was 50% A and 50% B for the analysis, with a flow rate of 500 μL/min.

Acyl-carnitines quantification was performed on the same samples by using a Varian Pursuit XRs Ultra 2.8 Diphenyl column (Agilent). Samples were analyzed by a 9 min run in positive ion mode. Mobile phases were A: 0.1% formic acid in H_2_O; B: 0.1% formic acid in MeOH; and the gradient was T0: 35% A, T2.0: 35% A, T5.0: 5% A, T5.5: 5% A, T5.51: 35% A and T9.0: 35% A, with a flow rate of 300 μL/min.

MultiQuant™ software (version 3.0.3, AB Sciex) was used for data analysis and peak review of chromatograms. Raw areas were normalized by the areas’ median. Obtained data were then compared to relative controls and expressed as fold change (FC). Obtained values were considered as scaled metabolite levels. Data processing and analysis were performed by MetaboAnalyst 5.0 web tool (Chong et al., 2019).

Statistical analysis for metabolomics data was performed by taking advantage of MetaboAnalyst 5.0 webtool using Student’s t-test for two group comparison (Chong et al., 2019).

The ATP energy charge ratio (Figure 2C) was calculated as: ([ATP]+0.5 [ADP])/([ATP]+[ADP]+[AMP]).

To measure glycerol release, cells (2.5 × 10^5^/well) were seeded in six well plate with 2 ml of medium. 48 h after, medium was collected and glycerol quantified by glycerol assay kit (Sigma-Aldrich, MAK117), following manufacturer instructions. Cells were counted and data normalized for cell number.

### 2.6 Confocal analysis

For LD analysis, cells were seeded on 13 mm coverslips and 24 h later treated over night with either DMSO, BAPTA-AM (10 μM, Sigma-Aldrich), U-73343 (2.5 µM, Sigma Aldrich), U-73122 (2.5 µM, Sigma-Aldrich), Etomoxir (20 μM, Sigma-Aldrich), Oleic acid (500 μM, Sigma Aldrich). After 16 h, cell were washed with PBS and fixed in 4% PFA in PBS solution for 10 min at RT, then washed (3 × 5 min) in PBS and incubated for 40 min with HCS LipidTOX™ Green Neutral Lipid Stain (Thermo Fisher H34475), according to the manufacturer’s instructions.

Images were collected with a Leica SP5 confocal microscope (DM IRE2) by WLL laser, exciting at 488 nm. Zoom, laser intensity, HyD gain were kept constant across different conditions for a better comparison. Images were analysed by Fiji (NIH), essentially as described (Rossini et al., 2021). Briefly, LD number, volume and surface was calculated through all stacks by the *3D Objects Counter* plugin after imposing a threshold (corresponding to 2× the mean fluorescence intensity of each cell), considering only objects in the range 5–500 pixels. The overall volume occupied by all LDs of each single cell was calculated to compare LD accumulation in the different experimental conditions.

For SREBP-2 immunofluorescence (IF), it was performed essentially as previously described (Romani et al., 2019), using α-SREBP-2 antibody 1:200 (Cayman, 10007663). Images were acquired sequentially with a Leica Stellaris confocal microscope equipped with Leica LAS X software. Typical acquisition settings were: image size 1,024x-1,024 pixels; acquisition mode xyz; pixel size 0.15μm; image depth 8 bits; acquisition speed 100Hz; Plan-Apochromat 63x/1.40 oil DIC M27 objective. For multichannel acquisitions, we used a main beam splitter 405/488/555/639. Raw images (saved in. czi or. lif formats) were opened in ImageJ and saved in exportable formats for analysis. Specific ROIs were traced in the nuclear region (stained with DAPI) to quantify the fluorescent signal (F) of SREBP-2.

### 2.7 Cell proliferation and migration assays

To evaluate cell proliferation upon different treatments, 1 × 10^5^ cells/well were seeded (t = 0 h) in six well plates and treated with either DMSO, BAPTA-AM (10 μM, Sigma-Aldrich), U-73343 (2.5 µM, Sigma Aldrich), U-73122 (2.5 µM, Sigma-Aldrich), Etomoxir (20 μM, Sigma-Aldrich). 24 h or 48 h later, cells were harvested and counted, normalizing their number to t = 0 h.

For cell migration, MDA-MB-231 CTRL and MDA-MB-231 MCU-KO cells were seeded at low confluency (30%) in 6-well plates. 24 h later they were treated either with DMSO, BAPTA-AM (10 μM, Sigma-Aldrich), U-73343 (2.5 µM, Sigma Aldrich), U-73122 (2.5 µM, Sigma-Aldrich), Etomoxir (20 μM, Sigma-Aldrich) or Oleic acid (500 μM, Sigma Aldrich) in 2% FCS medium. At the same time a linear scratch was obtained on cell monolayers through a vertically held P200 tip. Images were taken 24 h later. “TScratch” software (https://cse-lab.ethz.ch/software/) was used for automated image analysis.

### 2.8 Statistical analysis

All data are representative of at least 3 independent experiments. Significance was calculated by unpaired Student’s t-test for normally distributed and Wilcoxon Mann–Whitney test for not normally distributed data. When more than two groups were plotted for simplicity, only relevant pairwise comparisons were performed. * = *p* < 0.05, ** = *p* < 0.01, *** = *p* < 0.001. Values are reported as mean ± SEM.

## 3 Results

### 3.1 Both cytosolic and mitochondrial Ca^2+^ signaling are altered in TNBC cells

**FIGURE 1:**
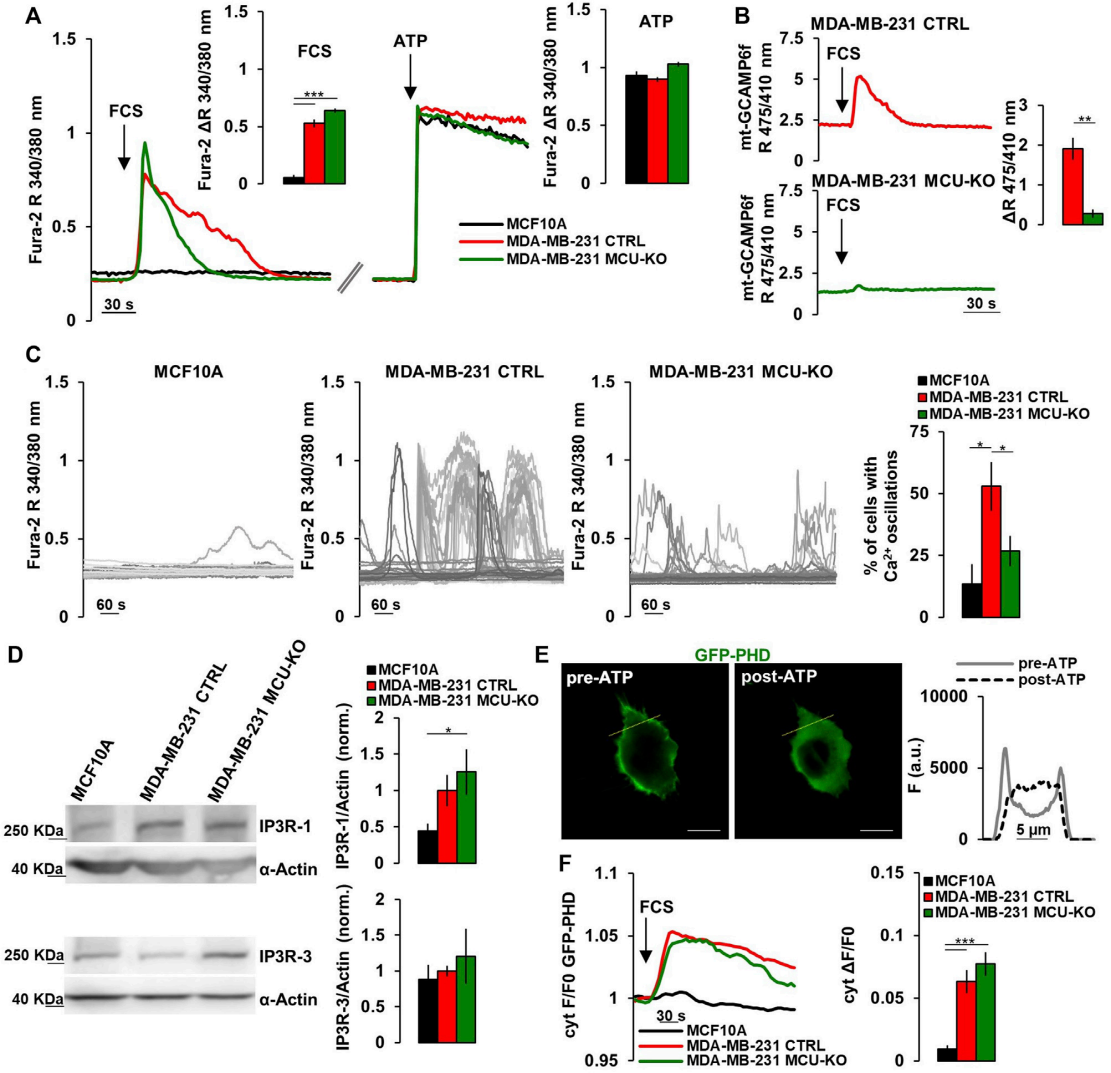
TNBC cells display frequent IP3-linked Ca^2+^ oscillations. **(A)** Representative traces of cytosolic Ca^2+^ measurements (expressed as FURA-2 340/380 nm ratio, R) in MCF10A, MDA-MB-231 CTRL and MDA-MB-231 MCU-KO cells upon acute stimulation (arrows) by FCS (2%) or ATP (100 µM). Bars represent the corresponding quantification of ΔR upon FCS or ATP stimulation, as indicated. n = 74–88 cells from 3 independent experiments. **(B)** Representative traces of mitochondrial Ca^2+^ dynamics (expressed as mt-GCAMP6f 475/410 nm R) in MDA-MB-231 CTRL and MDA-MB-231 MCU-KO cells upon acute stimulation (arrow) by FCS (2%). Bars represent the corresponding quantification of ΔR. n = 22–45 cells from 3 independent experiments. **(C)** Representative traces of cytosolic Ca^2+^ dynamics (measured as in (A)) in MCF10A, MDA-MB-231 CTRL and MDA-MB-231 MCU-KO cells, bathed in a solution containing FCS (2%). Each trace represents a cell. On the right, bars represent the percentage of cells displaying cytosolic Ca^2+^ oscillations within 10 min (see Methods). Mean ± SEM. n = 92–153 cells from 3 independent experiments. **(D)** Representative western blots (WB) and quantification of IP3R-1 and IP3R-3 expression levels (normalized to actin) in the indicated cell types. Mean +SEM. n = 4-7 independent experiments. **(E)** Representative image of a MDA-MB-231 CTRL cell expressing GFP-PHD, before and after ATP stimulation. On the right, traces represent the fluorescence intensity **(F)** along the linear region of interest (yellow ROI) in the images on the left, to visualize changes in signal distribution before and after ATP stimulation. Scale bar, 10 μm. **(F)** Representative traces (left) and quantification (right) of the fluorescence change (ΔF/F0) in the cytosol of the indicated cell types expressing GFP-PHD, upon acute stimulation with FCS (2%, arrow). Mean ± SEM. n = 21–30 cells from 3 independent experiments. *p < 0.05; **p < 0.01; ***p < 0.001.

It has been recently demonstrated that a constitutive, low-level IP3R-activity is crucial for cancer cell survival (Cardenas et al., 2016, 2020). Moreover, it has been reported that mitochondrial Ca^2+^ uptake fosters the invasiveness of TNBC cells (Tosatto et al., 2016). To further investigate the possible crosstalk between these pathways, we checked the amplitude of the cytosolic Ca^2+^ response in MCF10A cells (mammary epithelial, non-tumorigenic) and the TNBC cell line MDA-MB-231 (either control, CTRL, or MCU-KO (Tosatto et al., 2016)) upon acute exposure to fetal calf serum (FCS, which contains a mix of endogenous IP3-generating agonists (Cardenas et al., 2010; Rossi et al., 2020)). We found that MDA-MB-231 cells (both CTRL and MCU-KO) display a sustained cytosolic Ca^2+^ peak, whereas MCF10A cells do not (Figure 1A). Similar Ca^2+^ rises were observed in MDA-MB-231 cells bathed in Ca^2+^-free medium (Supplementary Figure S1A), suggesting that Ca^2+^ is mobilized from intracellular stores endowed with IP3-receptors (IP3Rs). As expected, we observed that FCS induces mitochondrial Ca^2+^ uptake in MDA-MB-231 CTRL, but not in MDA-MB-231 MCU-KO cells (Figure 1B). Importantly, the FCS-induced cytosolic Ca^2+^ peaks tend to last longer in MDA-MB-231 CTRL cells than in two different MCU-KO clones (Figure 1A; Supplementary Figure S1B), suggesting a crosstalk between cytosolic and mitochondrial Ca^2+^ dynamics and excluding possible clone-specific effects.

Notably, we found that MDA-MB-231 CTRL cells display frequent, spontaneous cytosolic Ca^2+^ oscillations when bathed in a medium containing FCS, whereas these events are blunted in two different MDA-MB-231 MCU-KO clones, and almost absent in MCF10A cells (Figures 1C; Supplementary Figure S1C). Similar spontaneous Ca^2+^ dynamics were observed also in MDA-MB-461 and BT-549 cells (Supplementary Figure S1D), suggesting this pattern is conserved in TNBC and not limited to MDA-MB-231 cells. Importantly, FCS-induced Ca^2+^ oscillations were also retrieved in mitochondria (Supplementary Figure S1E), suggesting these organelles sense and integrate cytosolic Ca^2+^ elevations. Overall, the above described differences between MCF10A and the MDA-MB-231 TNBC cell lines likely do not depend on substantial changes in the capacity of IP3Rs to release Ca^2+^ from the ER, because maximal IP3 generation upon ATP-mediated cell stimulation induces similar Ca^2+^ peaks in the 3 cell types (Figure 1A). Nevertheless, we found a tendency to a higher expression of IP3R-1, but not of IP3R-3, in MDA-MB-231 cells, compared to MCF10A (Figure 1D). Most importantly, by a previously reported sensor for phospholipase C (PLC)-δ1-activity (Hirose et al., 1999) (Figure 1E), we observed that FCS-stimulation induces a prompt PLC-activation (and thus IP3 generation) in MDA-MB-231, but not MCF10A cells (Figure 1F), thus explaining the different Ca^2+^ dynamics observed between TNBC and non-tumorigenic cells. On the other hand, similar IP3R-1/3 expression levels (Figure 1D) and PLC-activity (Figure 1F) were observed in MDA-MB231 CTRL and MCU-KO cells, suggesting that their different Ca^2+^ dynamics (Figures 1A, C) might depend on the complex crosstalk between cytosolic and mitochondrial Ca^2+^ oscillations (see discussion).

### 3.2 Mitochondrial Ca^2+^ signal modulates lipid metabolism in TNBC cells

**FIGURE 2:**
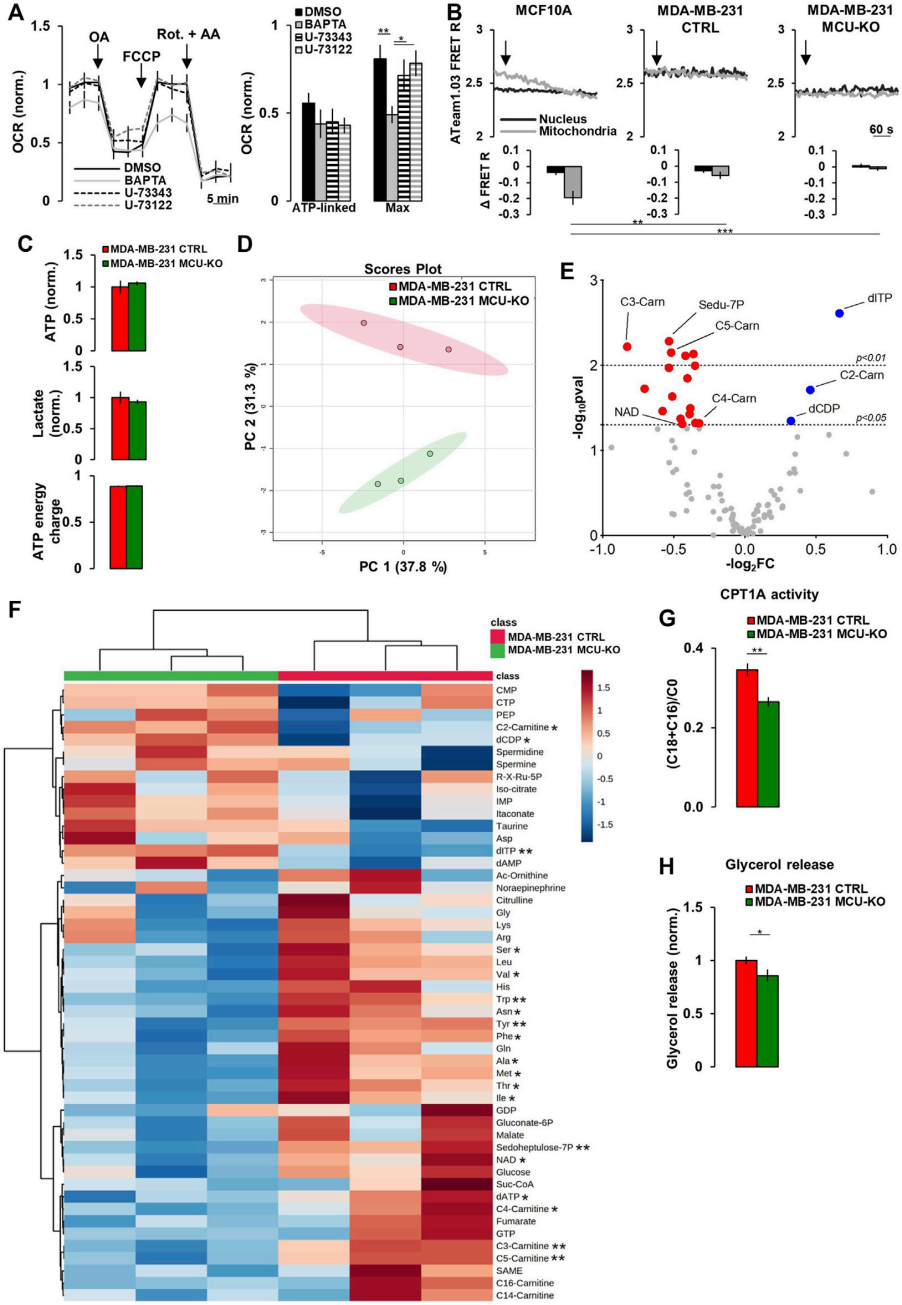
Mitochondrial Ca^2+^ signaling in TNBC cells regulates acyl-carnitine metabolism. **(A)** Traces represent mean OCR in MDA-MB-231 CTRL cells treated as indicated (OA = Oligomycin-A (1 µM); Rot. = Rotenone (1 µM); AA = Antimycin-A (1 µM); FCCP (1 µM)). U-73343 is an inactive analog of U-73122, used as control for this latter treatment. On the right, bars represent the ATP-linked and maximal OCR for each specific condition. Mean ± SEM. n = 9–15 wells from 3 independent experiments. OCR was normalized for cell number and for the basal OCR of the DMSO condition. **(B)** Representative traces of nuclear and mitochondrial ATP level variations (expressed as YFP/CFP FRET R) in the indicated cell types expressing nuclear and mitochondrial ATeam1.03 probes, upon inhibition of mitochondrial respiration by Rotenone (1 µM) + Antimycin-A (1 µM) (arrows). Bars represent the corresponding quantification, for each condition, of Δ FRET R after 10 min from Rot + AA addition. n = 18–23 cells from 3 independent experiments. **(C)** Bars represent the relative levels of ATP and lactate (normalized to those measured in MDA-MB-231 CTRL cells), or the ATP energy charge (see Materials and Methods) in the indicated cell types. Mean ± SEM. n = 3 independent experiments. **(D)** The scores plot shows the Principal Component Analysis to represent the distribution of the MDA-MB-231 CTRL and MDA-MB-231 MCU-KO cell group. n = 3 independent experiments/group. **(E)** Volcano plot of significantly increased (blue dots) and reduced (red dots) metabolites, mostly linked to acyl-carnitine pathway, in MDA-MB-231 MCU-KO cells, compared to MDA-MB-231 CTRL. Statistically significant metabolites were identified by Student’s *t*-test (log_2_FC≠0; -log_10_pval >1.3). n = 3 independent experiments. **(F)** Heatmap of the top 50 metabolites detected by mass spectrometry displaying the largest differences between MDA-MB-231 CTRL and MDA-MB-231 MCU-KO cells. Statistically significant metabolites were identified by Student’s *t*-test. **(G)** Bars represent the ratio between (C18:0 + C16:0)-acyl-carnitine and free-carnitine (C0) in the indicated cell types. Mean ± SEM. n = 3 independent experiments. **(H)** Bars represent the levels of glycerol (normalized to MDA-MB-231 CTRL cells) released in the growth medium of the indicated cell types. Mean ± SEM. n = 5-8 independent experiments. *p < 0.05; **p < 0.01; ***p < 0.001.

These constitutive, IP3-mediated Ca^2+^ whispers (that we here observed to be upregulated in TNBC cells) have been previously suggested to sustain mitochondrial metabolism and be critical for cancer cells survival (Cardenas et al., 2010, 2016, 2020; Filadi et al., 2018). However, the specific metabolic pathways regulated by this Ca^2+^ signal have not been fully elucidated. In MDA-MB-231 CTRL cells, the inhibition of FCS-induced Ca^2+^ oscillations, by either the intracellular Ca^2+^-chelator BAPTA or the PLC-inhibitor U-73122 (its inactive analog, U-73343, was used as control) (Supplementary Figure S1F), does not significantly affect ATP-linked oxygen consumption rate (OCR), though BAPTA reduces maximal OCR (Figure 2A). Moreover, in TNBC cells, the inhibition of mitochondrial respiration (by Rotenone/Antimycin-A treatment) does not significantly affect intracellular ATP levels, neither in the nucleus (which is in equilibrium with the cytosol (Imamura et al., 2009)), nor in the mitochondrial matrix (Figure 2B), suggesting that glycolysis might compensate and that the above described differences in Ca^2+^ dynamics do not significantly impact on ATP synthesis.

To identify the metabolic pathways regulated by mitochondrial Ca^2+^, we performed targeted metabolomics analysis in MDA-MB-231 CTRL and MCU-KO cells. The levels of ATP and lactate, as well as the ATP energy charge (see Materials and Methods), were similar between the 2 cell lines (Figure 2C), further suggesting that their different Ca^2+^ dynamics do not impinge on ATP turnover, nor the glycolytic rate. However, we found that the levels of different acyl-carnitines, as well as of several essential amino acids, are altered in MCU-KO cells (Figures 2D–F). We focused on the acyl-carnitine pathway, because MCU-ablation in skeletal muscle has been previously reported to deeply affect lipid metabolism (Gherardi et al., 2018). In particular, we noticed that the carnitine palmitoyl transferase 1A (CPT1A) activity index ((C18:0 + C16:0)/C0 (Audano et al., 2021)) is reduced in MCU-KO cells (Figure 2G). CPT1A catalyzes an essential step (*i.e*., the transfer of long-chain FA-CoA conjugates to carnitine) for the uptake of long-chain FA from the cytosol into mitochondria and their subsequent β-oxidation. In line with these data, the release of glycerol, a marker of triacylglycerol breakdown and FA oxidation by β-oxidation (Audano et al., 2021), is lower in MCU-KO cells (Figure 2H).

### 3.3 Lipid droplet turnover is altered in TNBC cells

**FIGURE 3:**
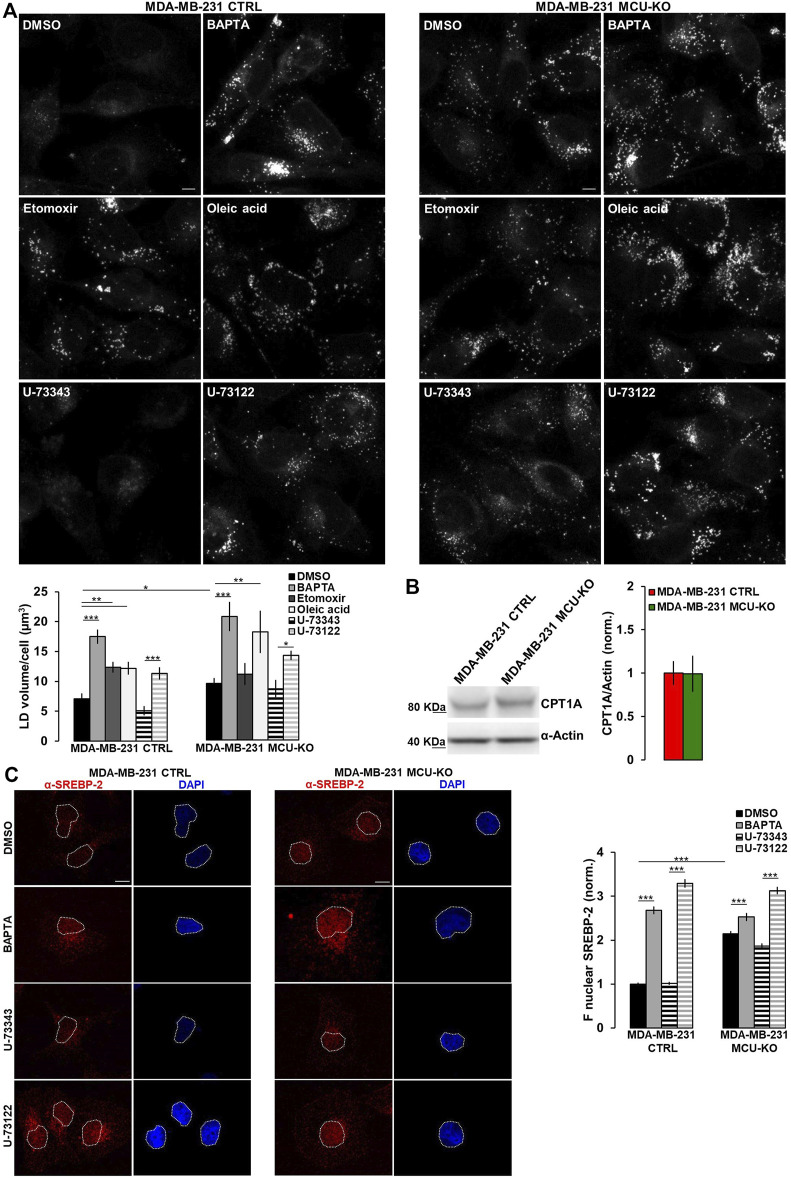
Ca^2+^ signaling in TNBC cells regulates LD turnover. **(A)** Representative confocal images (z-stack projection) of either MDA-MB-231 CTRL or MDA-MB-231 MCU-KO cells, treated as indicated and incubated with *LipidTOX stain* to visualize LDs (grey dots). Scale bar, 10 µm. Bars represent the overall volume of LDs for each cell in the indicated conditions. Mean ± SEM. n = 85–332 cells from 3 independent experiments. **(B)** Representative WB and quantification of CPT1A expression levels (normalized to actin) in the indicated cell types. Mean +SEM. n = 5-8 independent experiments. **(C)** On the left, representative confocal images of either MDA-MB-231 CTRL or MDA-MB-231 MCU-KO cells, treated as indicated and immunostained with a specific antibody for SREBP-2. Nuclei were stained by DAPI. Scale bar, 10 µm. On the right, bars represent the mean fluorescence intensity (F) of the SREBP-2 signal in the nuclear regions (highlighted by white ROIs in the corresponding images). Mean ± SEM. n = 90 cells from 3 independent experiments. *p < 0.05; **p < 0.01; ***p < 0.001.

Excessive free-FA are converted into neutral lipids and stored within lipid droplets (LDs). Notably, MDA-MB-231 CTRL cells do not show LD accumulation (Figure 3A). Conversely, treatment by etomoxir (a known CPT1A-inhibitor) leads to LD increase (Figure 3A). In line with a reduced CPT1A-activity, MDA-MB-231 MCU-KO cells show, compared to CTRL, a higher LD accumulation, similar to that induced by etomoxir in MDA-MB-231 CTRL, but not MCU-KO cells (Figure 3A). Importantly, Ca^2+^ oscillations are involved in this metabolic pathway, because their inhibition (either by BAPTA or U-73122 treatment) leads to LD accumulation in both MDA-MB-231 CTRL and MCU-KO cells (Figure 3A). Notably, no differences between these 2 cell types were observed anymore upon BAPTA or U-73122 treatment (Figure 3A), suggesting that cytosolic and mitochondrial Ca^2+^ oscillations coordinate LD turnover. As to the mechanism by which Ca^2+^ regulates FA metabolism, we observed similar expression levels of CPT1A in MDA-MB-231 CTRL and MCU-KO cells (Figure 3B). However, in the latter cell type, we detected a constitutive nuclear accumulation of the transcription factor sterol regulatory element binding protein 2 (SREBP-2), a key regulator of cholesterol and FA synthesis/metabolism (Bauer et al., 2011; Madison, 2016; Romani et al., 2019) (Figure 3C). Moreover, BAPTA, as well as U-73122 treatment, induces nuclear accumulation of SREBP-2 (Figure 3C), further suggesting that Ca^2+^ signaling might modulate FA metabolism and CPT1A activity by impinging on this pathway.

### 3.4 The Ca^2+^-mediated modulation of mitochondrial FA metabolism impinges on TNBC cell migration

**FIGURE 4:**
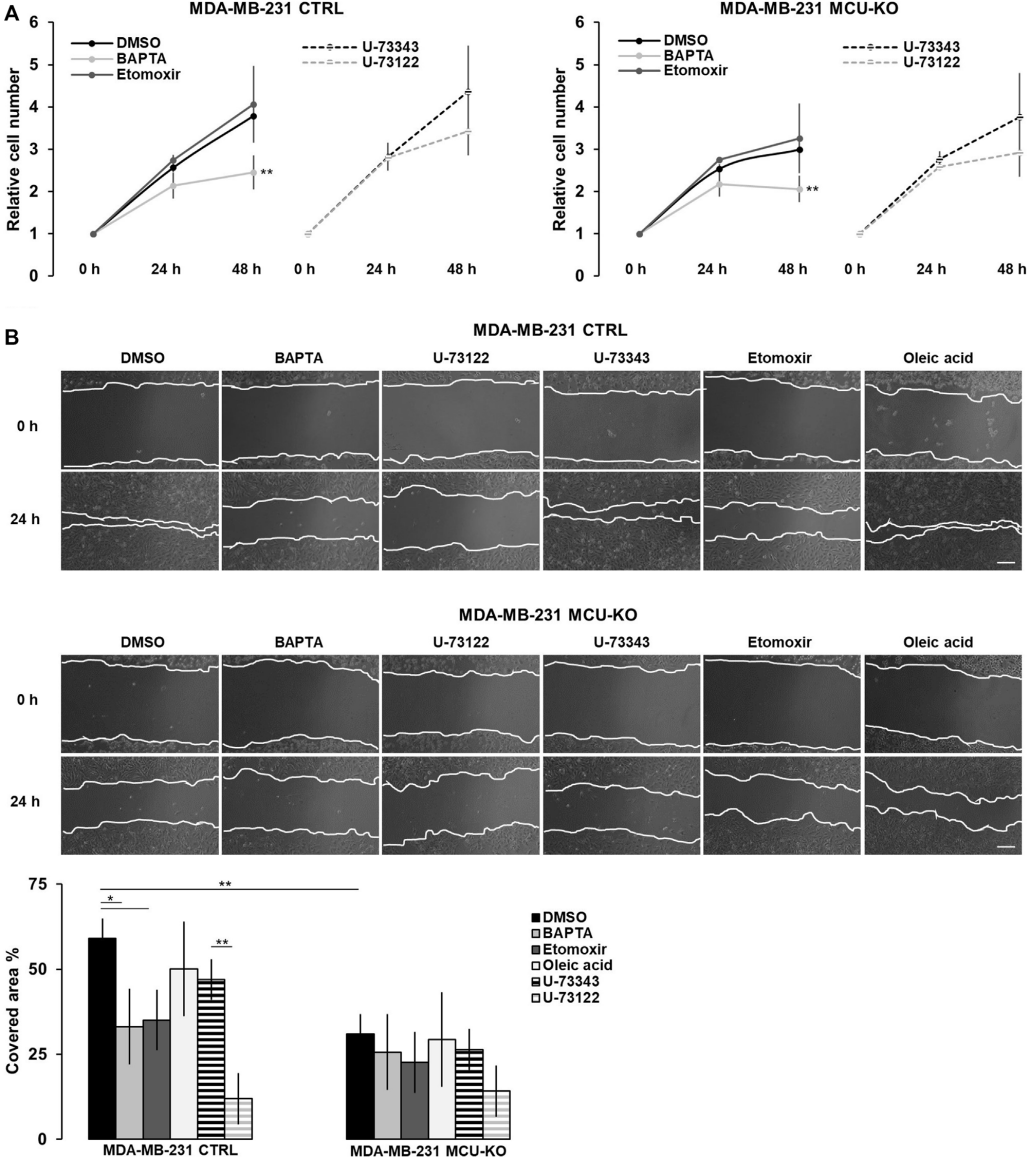
The Ca^2+^-FA metabolism axis modulates TNBC cell migration. **(A)** The relative cell number of either MDA-MB-231 CTRL or MDA-MB-231 MCU-KO cells, treated as indicated, was counted every 24 h for 2 days. Results were normalized for the number of cells at t = 0 h. Mean ± SEM. n = 5 independent experiments. **(B)** Representative images (top) and relative quantification (bottom) of the migration of MDA-MB-231 CTRL or MDA-MB-231 MCU-KO cells, treated as indicated. A linear scratch on the cell monolayer was traced a t = 0 h by a vertically held p200 tip. 24 h later, cell migration was calculated as percentage of the scratched area covered by cells. Mean ± SEM. n = 6–18 independent experiments. *p < 0.05; **p < 0.01.

Finally, we tested whether the Ca^2+^-mediated regulation of lipid metabolism affects proliferation and/or migration of TNBC cells *in vitro*. We found that only the complete ablation of cytosolic Ca^2+^ elevations by BAPTA slightly reduces cell proliferation in both genotypes (CTRL and MCU-KO, see also (De Mario et al., 2021)), whereas CPT1A inhibition has not effect (Figure 4A). Conversely, both the inhibition of Ca^2+^ oscillations (either by BAPTA or U-73122 treatment) and CPT1A activity reduce cell motility in MDA-MB-231 CTRL, but not MDA-MB-231 MCU-KO cells (Figure 4B). Of note, in line with previous observations (Tosatto et al., 2016), cell motility is lower in MDA-MB-231 MCU-KO cells, compared to MDA-MB-231 CTRL (Figure 4B). Importantly, the accumulation of LDs observed upon inhibition of Ca^2+^ oscillations (Figure 3A) does not regulate cell motility *per se*, because a similar accumulation induced by a different, Ca^2+^- and CPT1A-independent treatment (*i.e.*, oleic acid, Figure 3A) does not significantly affect this parameter (Figure 4B). Overall, these data suggest that cytosolic and mitochondrial Ca^2+^ signals cooperatively modulate CPT1A activity and mitochondrial FA uptake, in turn sustaining cell migration by an unknown mechanism (see discussion).

## 4 Discussion

Accumulating evidence suggests that rewiring of mitochondrial metabolism is critical in several cancers, with continuous adaptations accompanying tumor progression. The use of TCA cycle substrates alternative to pyruvate to supply anaplerosis, particularly glutamine (glutaminolysis), has been reported in several malignancies characterized by a defective OXPHOS (Altman et al., 2016; Mullen et al., 2012). Intriguingly, beyond their role in cell bioenergetics/biosynthesis, several TCA cycle-associated molecules (including succinate, fumarate, itaconate, D/L-2-hydroxyglutarate (2-HG), acetyl-CoA) are endowed with key signaling functions (Ryan et al., 2019; Yong et al., 2020). For instance, metabolic changes in cancer are frequently associated with epigenetic alterations, such as histone/DNA methylation (enhanced by the accumulation of fumarate, succinate and 2-HG) or histone acetylation (regulated by acetyl-CoA availability) (Ryan et al., 2019; Yong et al., 2020). Remarkably, cancer-associated mutations in some TCA cycle enzymes, including fumarate hydratase (FH, linked to hereditary renal cell carcinomas, RCC), succinate dehydrogenase (SDH, linked to paraganglioma) and isocitrate dehydrogenases (IDH, linked to glioma and acute myeloid leukemia, AML) dictate specific metabolic patterns by altering the accumulation of these signaling intermediates (Yong et al., 2020). Though most cancer cell types produce ATP by upregulating glycolysis, their reliance on OXPHOS increases in advanced stages of the disease (Faubert et al., 2020), with cellular invasion, migration and metastasis correlating with enhanced respiration and mitochondrial biogenesis in breast cancer (Lebleu et al., 2014).

Of note, several mitochondrial activities are regulated by Ca^2+^ signals (Rossi et al., 2019), suggesting the intriguing possibility that Ca^2+^ signaling might be exploited to rewire mitochondrial metabolism. In the mitochondrial matrix, Ca^2+^ positively modulates the activity of three TCA cycle enzymes, while in the intermembrane space it stimulates different metabolite transporters (Rossi et al., 2019), thus sustaining OXPHOS. Conversely, an excessive mitochondrial Ca^2+^ influx can promote ROS generation, Ca^2+^ overload, mitochondrial permeability transition pore opening and cell death (Rossi et al., 2019). Different oncogenes reduce ER Ca^2+^ content and/or inhibit its release through IP3Rs, thereby dampening excessive ER to mitochondria Ca^2+^ transfer, lowering pro-apoptotic mitochondrial Ca^2+^ uptake and favouring cancer cell survival (Chen et al., 2004; Rimessi et al., 2020). Consistently, several tumour suppressors exert opposite effects (Giorgi et al., 2010; Bononi et al., 2017).

However, recent studies reappraised this notion. In breast cancer samples, MCU expression positively correlates with tumor progression, suggesting that mitochondrial Ca^2+^ uptake could be advantageous for cancer growth and metastasis (Tosatto et al., 2016). An upregulated constitutive, low-level, IP3R-mediated mitochondrial Ca^2+^ uptake has been suggested to be essential for cancer cell survival by sustaining TCA cycle, thus allowing to cope with high energy demand and the huge need of metabolic substrates (in particular for nucleotide synthesis (Cardenas et al., 2016)). The suppression of this inter-organelle Ca^2+^ whisper is tolerated in normal, but not in cancer cells (Cardenas et al., 2010, 2016; Filadi et al., 2018), suggesting a therapeutic opportunity. On this line, the expression of different IP3R isoforms (possibly feeding basal mitochondrial Ca^2+^ signals) is upregulated in several malignancies and is critical in epidermal growth factor-induced epithelial-mesenchymal transition (EMT) (Rosa et al., 2020). In diffuse large B-cell lymphoma and chronic lymphocytic leukemia cells, an upregulation of pro-survival, basal IP3-dependent Ca^2+^ signaling has been reported (Bittremieux et al., 2018).

Here, we investigated whether and how Ca^2+^ signaling contributes to the rewiring of mitochondrial metabolism in TNBC cells, as well as to their invasiveness. We found that TNBC cells display frequent, spontaneous IP3-linked cytosolic Ca^2+^ oscillations sensed by mitochondria, with mitochondrial Ca^2+^ uptake further sustaining these events. Indeed, we observed that MCU ablation substantially reduces their frequency, though the precise mechanism by which mitochondria tune this parameter is not completely clear. Yet, the effect might be linked to the reported capacity of mitochondria to shape cytosolic Ca^2+^ dynamics, which relies on a complex crosstalk between mitochondrial Ca^2+^ uptake and buffering, the subsequent mitochondrial Ca^2+^ efflux, and the interference of these opposite processes with the Ca^2+^-mediated modulation of both IP3R and PLC activity (Landolfi et al., 1998; De Stefani et al., 2011; Rizzuto et al., 2012; Filadi et al., 2017). For instance, IP3R activity is modulated either positively or negatively by respectively mild or high Ca^2+^ elevations. In MDA-MB-231 CTRL, but not MCU-KO cells, mitochondrial Ca^2+^ uptake/buffering could dampen the high Ca^2+^ concentration reached near the mouth of IP3R, avoiding a premature inactivation of the channel inevitably leading to the termination of the Ca^2+^ spike. Moreover, after uptake, mitochondrial Ca^2+^ efflux could contribute in keeping, at ER-mitochondria interface, the right window of Ca^2+^ concentrations to further sensitize IP3R opening, prolonging (see the different kinetic in Figure 1A) and/or inducing repetitive Ca^2+^ oscillations.

Nevertheless, we found that this signaling pathway regulates the uptake of long chain FA into mitochondria, a process strictly controlled by CPT1A activity. To the best of our knowledge, a direct regulation of CPT1A by Ca^2+^ has not been reported. However, it has been recently demonstrated that sustained cytosolic Ca^2+^ levels, upon ER Ca^2+^ release and activation of store-operated Ca^2+^ entry (SOCE), regulate the mobilization of lipids from LDs and FA oxidation (FAO) by controlling the expression and activity of different lipases (Maus et al., 2017). Specifically, the mechanism involves the Ca^2+^-dependent activation of adenylyl cyclases and elevation of cyclic adenosine monophosphate (cAMP) levels, in turn activating specific lipases by protein kinase A-mediated phosphorylation and regulating lipid metabolism through the CREB-PPARα-PGC1α transcriptional pathway (Maus et al., 2017). Considered the frequent cycles of IP3-dependent ER Ca^2+^ release here reported in TNBC cells, which are very likely associated with SOCE activation, we hypothesize that a similar mechanism might be in place, with a Ca^2+^-mediated control of lipase activity and of specific, lipid metabolism-associated transcriptional pathways. Remarkably, a hint of this latter mechanism is represented by our finding that SREBP-2 is activated upon inhibition of Ca^2+^ oscillations, though the mechanistic details remain to be investigated.

Noteworthy, beside the mechanism, our data suggest that the Ca^2+^-regulated mitochondrial FA uptake is critical to sustain TNBC cell migration, because the inhibition of this pathway by different approaches triggers LD accumulation (as consequence of the reduced CPT1A activity) and affects cell invasiveness. Importantly, LD accumulation is a consequence of the reduced mitochondrial FA uptake, but does not dampen cell migration *per se*. This suggests that the critical step regulating motility might be the metabolic reactions following mitochondrial FA uptake, including FAO. FA turnover is known to be critical for *de novo* membrane synthesis, as well as to generate key intermediate molecules endowed with signaling activity, highlighting how this metabolic pathway might regulate tumor metastasis (Röhrig and Schulze, 2016). For instance, the uptake, utilization and enhanced intracellular trafficking of adipocyte-derived FA has been shown to promote breast cancer progression (Yang et al., 2018), as well as to be critical in colon cancer, whereby CPT1A-dependent FAO upregulates Wnt/β-catenin signaling by regulating β-catenin acetylation (Xiong et al., 2020). Remarkably, FAO has been shown to underlie the activation of Src oncoprotein in TNBC, promoting metastasis (Park et al., 2016). Further investigations will be necessary to precisely understand how Ca^2+^ signaling regulates FA metabolism, thus controlling TNBC invasiveness.

Overall, our findings suggest that TNBC progression is regulated by sustained IP3-linked Ca^2+^ oscillations, in line with the recent evidence suggesting that mitochondrial Ca^2+^ addiction is a feature of certain cancer cells (Cardenas et al., 2016, 2020; Tosatto et al., 2016; Bittremieux et al., 2018). Given the Ca^2+^ gamut of capabilities, it is possible that subtle alterations in different Ca^2+^ signaling pathways may account for TNBC-associated metabolic alterations. Although it is unlikely that these latter are solely mediated by Ca^2+^ signals, yet it will be of immense help to define the mechanisms underlying the here reported crosstalk between mitochondrial Ca^2+^ signaling and FA metabolism in TNBC cells. We envisage this step will be critical to designing specific pharmacological interventions and determining whether Ca^2+^ modulation can be harnessed as a novel therapeutic opportunity to treat TNBC.

## Data Availability

The original contributions presented in the study are included in the article/Supplementary Material, further inquiries can be directed to the corresponding author.
