# Supplementary material for: Sustained IP3-linked Ca2+ signaling promotes progression of triple negative breast cancer cells by regulating fatty acid metabolism
Source: Front Cell Dev Biol. 2023 Mar 13;11:1071037. doi: 10.3389/fcell.2023.1071037 (PMC10040683; doi:10.3389/fcell.2023.1071037)
Supplement: Supplementary file 1 [file DataSheet1.pdf]

Supplementary Figure 1

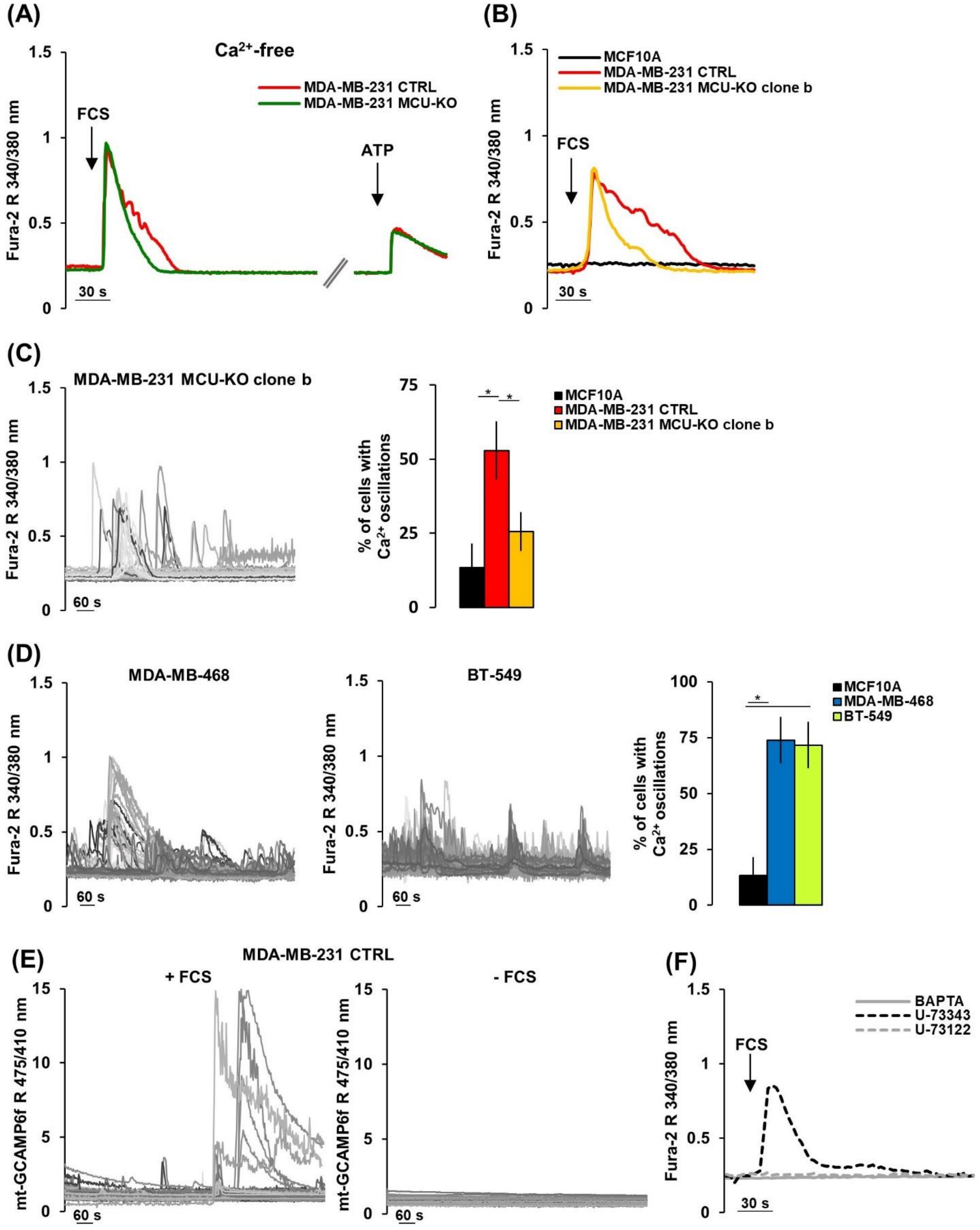

Supplementary Figure 1. Ca^{2+} dynamics in TNBC cells. (A) Representative traces of cytosolic Ca^{2+} dynamics (expressed as FURA-2 340/380 nm R) in MDA-MB-231 CTRL and MDA-MB-231 MCU-KO cells bathed in a Ca^{2+}-free, EGTA-containing medium, upon acute stimulation (arrows) with FCS (2%) or ATP (100 µM). n = 25-48 cells from 3 independent experiments. (B) Representative traces of cytosolic Ca^{2+} variations (measured as in Figure 1A) for the indicated cell types upon acute stimulation with FCS (2%). The traces of MCF10A and MDA-MB-231 CTRL cells are the same of

Figure 1A for a better comparison. n = 72-106 cells from 3 independent experiments. (C) Representative traces of cytosolic Ca^{2+} dynamics (measured as in Figure 1C) in MDA-MB-231 MCU-KO clone b cells (see methods), bathed in a solution containing FCS (2%). Each trace represents a cell. n = 112 cells from 3 independent experiments. On the right, bars represent the percentage of cells displaying cytosolic Ca^{2+} oscillations within 10 min (see Methods) for the indicated cell type. The bars of MCF10A and MDA-MB-231 CTRL cells are the same of Figure 1C for a better comparison. Mean ± SEM. n = 92-153 cells from 3 independent experiments. (D) Representative traces of cytosolic Ca^{2+} dynamics (measured as in Figure 1C and S1C) in MDA-MB-468 and BT-549 cells, bathed in a solution containing FCS (2%). Each trace represents a cell. n = 99-107 cells from 4 independent experiments. On the right, bars represent the percentage of cells displaying cytosolic Ca^{2+} oscillations within 10 min (see Methods) for the indicated cell type. The bar of MCF10A is the same of Figure 1C for a better comparison. Mean ± SEM. n = 92-107 cells from 4 independent experiments. (E) Representative traces of spontaneous mitochondrial Ca^{2+} dynamics (expressed as mt-GCAMP6f 475/410 nm R) in MDA-MB-231 CTRL cells bathed in a solution either containing (left, +FCS) or not (right, -FCS) FCS (2%). n = 53-67 cells from 4 independent experiments. (F) Representative traces of Ca^{2+} dynamics measured by Fura-2 in MDA-MB-231 CTRL cells, treated overnight with either BAPTA-AM (10 µM), U-73343 (2.5 µM) or U-73122 (2.5 µM), upon acute exposure to FCS (2%). n = 32-63 cells from 3 independent experiments. *$p<0.05$.
